# Perceived strategies to reduce traumatic childbirth amongst Iranian childbearing women: a qualitative study

**DOI:** 10.1186/s12884-020-03045-0

**Published:** 2020-06-08

**Authors:** Mahshid Taheri, Ziba Taghizadeh, Nahid Jafari, Amirhossein Takian

**Affiliations:** 1 Midwifery Office, Ministry of Health and Medical Education, Tehran, Iran; 2 Department of Reproductive Health, School of Nursing and Midwifery, Tehran University of Medical Sciences, Tehran, Iran; 3 Department of Primary Healthcare, Ministry of Health and Medical Education, Tehran, Iran; 4 Department of Health Management and Economics, School of Public Health, Tehran University of Medical Sciences, Tehran, Iran; 5 Department of Global Health and Public Policy, School of Public Health, Tehran University of Medical Sciences, Tehran, Iran; 6 Health Equity Research Centre (HERC), Tehran University of Medical Sciences, Tehran, Iran

**Keywords:** Psychological birth trauma, Prevention, Maternity care, Primary healthcare, Iran

## Abstract

**Background:**

Psychological birth trauma (PBT), mainly due to overlooking maternal mental health, is a common and high prevalence public health problem in low-resource settings. Preventing PBT is a good indicator of the realization of human rights in healthcare. This work reports the results of a qualitative study that aimed to identify perceived strategies of PBT prevention among childbearing women in Iran.

**Methods:**

We conducted semi-structured in-depth interviews with 22 mothers with history of traumatic childbirth, two mothers with positive childbirth experience, two spouses, and eight health professionals between April and June 2017. We used purposive sampling method to recruit traumatized mothers, while health experts were selected based on their relevant expertise and experience. Our initial literature review identified eight categories, using which we developed our interview guide and conducted the content analysis approach.

**Results:**

With the maximum possible purification, we reached 50 thematic codes. The strategies to prevent PBT are generally summarized in four major themes and 13 categories: 1) skill-builder knowledge [Birth preparedness, Mothers’ empowerment in maintaining mental health, Understanding the importance of mental care in maternity services], 2) responsible caregiving [Support loop, Good behavior of the caregivers, Deepening trust, Struggle with medicalization of childbirth, Labour pain relief, Special services for maternal mental health], 3) the alliance of prenatal and antenatal care [Continuity of care, Coordination of prenatal and antenatal caregivers], and 4) reconstruction of the structures [Efficient management, Rebuilding physical structures].

**Conclusions:**

This is a comprehensive approach towards PBT prevention, which can guide future efforts to reduce PBT at the clinical level and open further avenues for future studies. We recommend policy makers to consider the integration of multilevel and multidimensional PBT prevention interventions, simultaneously within maternity care services packages for promotion of mental health.

## Background

Psychological birth trauma (PBT) has been defined as a mental distress caused by actual or threatened injury to mother or her newborn that may occur during the labour or birth. PBT might interfere with mother’s normal response to the birth stress [1]. Trauma exposure and negative experience of birth care is associated with post-traumatic stress disorder (PTSD) following PBT [2]. A United States-based study reported the incidence of PBT as 34%; 34.3% of those experiencing trauma, had some PTSD symptoms and 5.7% were fully symptomatic [3]. 54.5% of women in Iran reported their birth experiences as traumatic [4]. A-criterion of “Diagnostic and Statistical Manual of Mental Disorders, 5th edition (DSM-V) is the golden standard for diagnosing PBT [5, 6].

Prevention of mental suffering is an important means to progress towards human development and the realization of human rights in healthcare [7]. The published studies on PBT have mostly addressed its prevalence, risk factors, complications and treatment. As our previous literature reviews revealed, a hitherto poorly addressed issue in the PBT literature is the identification of direct interventions to prevent PBT [8]. Despite some evidence-based strategies and potential approaches to prevent PBT, the research is lacking in this area and an information gap exists about the listed strategies that can cover all aspects of maternity care and result in clinical success [9]. A comprehensive approach is therefore necessary for successful prevention of mental illnesses; which requires interdisciplinary efforts through multilevel and multidimensional strategies [7].

Good quality birth care, positive attitude of healthcare providers, and high quality midwifery skills are the prerequisites for a desirable childbirth experience that can prevent PBT. A national action plan that is based on all possible PBT prevention strategies [10], may act as a multi-purpose solution to reduce the high incidence of PBT and its permanent psychological complications in low-resource settings [11].

Currently, due to the over-medicalization of childbirth, Iran has the world’s second highest rate of Cesarean section (CS) [12, 13]. Specialist Obstetricians are the main caregivers for healthy pregnant women in Iran, while midwives’ autonomy is limited in labor wards. Chronic lack of preparatory birth measures has led many pregnant women to fear normal delivery. Birth companions during labor are only allowed in some hospitals, which along with general lack of support, may negatively affect the mothers’ mental health. Pain relief methods are not applicable for normal delivery in many public hospitals. High expenses and shortage of human resources are perceived to be the main reasons behind these problems [13]. As an initial attempt to create national PBT prevention plan, this article reports the perceived strategies to prevent PBT among childbearing women in Iran. Our findings and policy recommendation can help, we envisage, ongoing policies to increase the safe normal delivery using high-quality maternity care in Iran and beyond.

## Methods

### Design

We collected data during two phases. During the first phase, we conducted a comprehensive literature review; i.e. electronic data sources, international and national guidelines and the related NGOs; to identify all available interventions and categorize the effective strategies to reduce PBT. Studies were appraised systematically using Cochrane risk bias tool; bias is checked qualitatively from seven domains including random sequence generation, allocation concealment, participants and personnel blinding, assessment blinding, addressing incomplete outcome data, selective reporting, and other sources of bias. The extracted data were summarized and the effective strategies were categorized. The second phase aimed to develop the available strategies, that were found in the first phase. This manuscript describes the second phase in detail and reports the final results of both phases. We used a directed qualitative content analysis to explore, summarize, and classify the perceived strategies to reduce PBT. It is recommended to use this deductive approach if a phenomenon is incomplete or needs further description [14].

### Sampling and recruitment

We recruited 34 participants from two different population groups: 22 mothers with history of PBT and eight health professionals: three clinical practitioners, three policy makers and two faculty members. Two husbands of the traumatized mothers and two mothers with the positive birth experience were other key groups. We purposefully recruited the mother informants from selected public primary healthcare centers in the capital city of Tehran. Our participants should have given birth in the last two years, as well as express negative birth experience (NVD or C/S). Based on A-criterion of post-traumatic stress disorder, mothers with PBT had two signs in face to face interview [6]:

- Had experienced an event that involved actual or threatened death or injury for themselves or their baby during the childbirth.
- Their response about childbirth involved intense fear, helplessness, or horror.

Health expert participants were selected based on their relevant expertise and experience; they were sampled from the Ministry of Health and Medical Education (MOHME), three hospitals and two Universities of Medical Sciences in Tehran. Current severe psychiatric disorder, e.g. psychosis was an exclusion criterion. The maximum variation sampling was considered regarding age, educational background, employment status, parity and the mode of delivery. An estimation of the needed sample size was determined as sufficient for analyzing the main themes. Table 1 presents demographic characteristics of study participants. According to the self-statement of traumatized mothers, the main reason for PBT included “emergency cesarean section” (nine cases), “the risk of complication for baby” (five cases), “helplessness” (five cases), and “the risk of death for herself” (three cases).

**Table 1:** Demographic Characteristics of Respondents (*N* = 34)

Variable	Mothers (*N* = 24)	Husbands (*N* = 2)	Experts (*N* = 8) 1 male, 7 females
Age range (Mean)	18–40 (27.2)	32 and 47 (39.5)	38–60 (51.7)
Education status			
Primary education	5		
High school graduate	9		
Bachelor degree	6	1	1
Master degree	3		1
Doctorate degree	1	1	6
Employment status			
None	13		
Full time	9	2	8
Part time	2		
Type of delivery			
Vaginal	8		
Cesarean	16		
Birth attendant			
Midwife	5		
Obstetrician	19		
Type of hospital			
Private	7		
Public	17		
Pariety			
Nullipara	11		
Multipara	13		

### Procedure

We obtained written informed consents from all eligible participants and conducted face-to-face semi-structured in-depth interviews (30–60 min) that was digitally recorded. Interviews with mothers were conducted by the second author (ZT) in a private room inside the health centers while interviews with experts were carried out by the first author (MT) in the private offices of the interviewees. Initially, we gathered some demographic information, and subsequently moved to several open-ended questions that were prepared based on the literature (Table 2). Probing questions were asked when more clarification was needed. Interviews were performed from April to June 2017. All interviews were transcribed verbatim and analyzed.

**Table 2:** The generic semi-structured interview guide

Questions for mothers	
• What was the main cause for your negative birth experience? • Was there a way that you would not be traumatized psychologically? • What could you do to help yourself in prenatal or antenatal period? • How could your carers prevent this trauma? • What is your solution to prevent trauma in other childbearing women? • What should we do to not be traumatized?	
Questions for experts	
• What is your suggested solution to minimize the traumatic childbirth in our hospitals? • Is there any prevention way before labour? • What is the role of mother, her partner and caregivers in this prevention? • What are the shortages of our maternal care in this area?	
Questions for husbands	
• From your point of view, what was the main cause for your partner’s traumatic birth? • What could you do to help your partner in prenatal or antenatal period? • How could maternity carers prevent this trauma?	

### Data analysis

We employed MAXQDA software version 10 to facilitate data management and coding process. We read transcripts several times. The first (MT) and third authors (NJ) conducted thematic analysis using directed content analysis, which was approved by the senior author [14]. Our systematic review, which was conducted prior to this qualitative study, identified eight categories for prevention of PBT (Table 3), which guided our initial coding. We broke the transcripts down into the smallest meaningful units (preliminary codes). Using the deductive approach, the codes were compared according to the familiarity of the content. The codes that were in line with one of predefined eight categories were placed within their sub-categories, while ones with new contents led to emergence of new categories. As the number of codes increased, new sub-categories were formed and each category included several subcategories. Reworking data established links between sub-categories and main categories. The final step was the abstraction of themes from main categories. An example of the analysis process is given below:

**Table 3:** Predefined categories extracted from the literature review

**Categories**	
*Birth preparedness*	
*Supportive loop*	
*Good behavior of the caregivers*	
*Deepening trust*	
*Struggle with medicalization of childbirth*	
*Labour pain relief*	
*Coordination of prenatal and antenatal caregivers*	
*Continuity of care*	

‘Mother should know all options, potential complications and alternatives during labour. Details of childbirth care and interventions must be given to her in written form during prenatal visits.’ (text) → Being prepare for all possible options during labour (preliminary code) → Birth plan preparation (sub-category) → Birth preparedness (main category) → Skill-builder knowledge (theme).

We ensured credibility of this study through continuous involvement of the participants; as they checked the initial codes of their interviews before finalizing categorization. The coherence of the final results was verified by three participants (two experts and one mother). In order to achieve a consensus in coding, peer checks were performed by the entire research team led by the senior author.

### Ethical considerations

This research was approved by the ethical committee of Tehran University of Medical Sciences (IR.TUMS.VCR.REC.1395.374). Written informed consent was signed voluntarily by the participants. Confidentiality of the collected data was promised. Participants were informed about their right to withdraw at any stage of the interview. Recorded voices were destroyed after analysis.

## Results

Our content analysis resulted in the extraction of 739 codes. This qualitative study added a lot of details to the pre-defined categories; which are indicated with an asterisk (*) in the text and Table 4. With the maximum purification possible, 49 strategies were obtained; of which 34 strategies belonged to the predefined categories and 15 strategies with the new contents that resulted in the emergence of five new categories. These 13 (eight from the first phase and five from the second phase) categories were organized under four themes: skill-builder knowledge, responsible caregiving, the alliance of prenatal and antenatal care, and reconstruction of the structures. Skill builder knowledge includes 11 strategies that can prepare, physically and mentally, mothers for childbirth or modify care providers’ attitude. Responsible caregiving includes 24 strategies that may lead to high quality childbirth care. The alliance of prenatal and antenatal care refers to the six strategies of continuity of care or its alternatives. Reconstruction of the structures includes eight strategies regarding maternity care reforms and modernization of hospitals. Table 4 represents the details of categorized strategies.

**Table 4:** Themes, categories and perceived strategies to reduce PBT amongst childbearing women

Skill-builder knowledge	Responsible caregiving	The alliance of prenatal and antenatal care	Reconstruction of the structures
***Birth preparedness*** 1. Providing realistic information about childbirth 2. Birth adjustment skills training 3. Upgrading the quality of childbirth courses 4. Birth plan preparation 5. Encouraging mothers to take part in childbirth courses* 6. Setting up virtual childbirth courses 7. Reproductive health education to different age groups* Mothers’ empowerment in maintaining mental health* 1. Self-care for mental health* 2. Promoting Spiritual Health* Understanding the importance of mental care in maternity services* 1. Enhancing medical education in mental health care* 2. In-service training regarding maternal mental health care*	***Support loop*** 1. Birth companion 2. Caregivers’ supportive behavior 3. Supporting through one to one care 4. Special support in emergency situations 5. Gaining support from mother’ s social networks during pregnancy 6. Providing conditions for support from peers* ***Good behavior of the caregivers*** 1. Good-humored health staff 2. Respectful maternity care 3. Knowledge and prosperity are the cornerstone of good behavior* ***Deepening trust*** 1. Getting familiar with the birth attendant during prenatal period 2. Getting mother’s trust from the first encounter 3. Providing timely information for mother 4. Paying attention to the mother’s needs ***Struggle with medicalization of childbirth*** 1. Early labour assessment 2. Promoting physiologic childbirth* 3. Midwifery-based care 4. Separating maternity and gynecologic care* ***Labour pain relief*** 1. Relaxing through labour massage 2. Listening to the self-selected music during labour 3. Freedom of movement in birth places 4. Accepting the labour pain* Special services for maternal mental health* 1. Setting standards for mental health care of pregnant women* 2. Special interventions for high-risk mothers* 3. Implementation of prenatal screening program for mental health problems*	***Continuity of care*** 1. Continuity of care by the same midwife(s) from prenatal to postpartum 2. Supporting the private sector of maternity care* ***Coordination of prenatal and antenatal caregivers*** 1. Coordinating the goals of prenatal and childbirth care* 2. National electronic prenatal-care records system* 3. Setting the regional referral system to connect health-centers and hospitals* 4. The clarity of prenatal records for birth attendants	Efficient management* 1. The adoption of new laws* 2. Monitoring the implementation of laws* 3. Financing the personnel* 4. The use of human resources* 5. The appropriateness of the description of tasks with the capacity of labor* 6. Scoring system for health settings* Rebuilding physical structures* 1. Creating an inviting labour wards* 2. Covering any visible hospital equipment*

* Strategies or categories that were not pre-defined and obtained directly from qualitative study

### Skill-builder knowledge

Information that can potentially create or reinforce a person’s skills is called Skill-builder knowledge. The strategies of this theme are training the mother and the medical staff. The 70 interview codes referred to strategies that will improve the mother’s skills in adjusting to the labour process through education:

*“I was not ready for childbirth. I did not even know when to go to the maternity hospital. Everything was new there, and I was afraid of every move as I didn’t know anything”.*

One clinician confirmed that “*besides the fact that many of our mothers receive no special training for childbirth, some childbirth classes’ efficiency is very poor and superficial. This is why mothers are horrified at labor - we need practical training”.*

A group of strategies of this theme referred to mothers’ psychosocial empowerment training*, as most traumatized mothers lacked enough skills to maintain their mental health:

*“Maternal mental health can be promoted through special education. Prenatal training should include skills for effective communication, coping with stress, managing interpersonal relationships, and decision making in critical situations”.*

One strategy: “pregnancy and childbirth caregivers should be trained to gain mental care skills*”, was frequently mentioned to improve the situation:

‘*We just dealt with the body. A doctor is so unaware of the psychological issues saying directly to the mother's face that he sees two cysts in the infant's brain - the trauma that this has imposed on the mother cannot be erased so easily’*.

### Responsible caregiving

The most important PBT prevention strategies fall into this theme. Clinical caregivers can reduce the likelihood of PBT by modifying their approach and changing the way(s) that maternity care is provided. Five out of the six categories of this theme were identified prior to our content analysis, most of which were also repeated by the interviewees. The support loop was a category that was expanded during content analysis through 177 codes. Supporting mother was identified as the most valuable evidence-based approach in preventing childbirth trauma, which is provided by the birth companion, relatives of the mother, caregiver staff, and peers in group care*, all of whom can be accessed through direct supervision and guidance of the one responsible for mother’s care. One of the most important needs of traumatized mothers is protection in emergency situations, such as preterm delivery or the birth of a baby under distress, which was mentioned by many mothers:

“*For the baby, they told me I had to go to the operating room and I felt so bad. They gave no explanation and just said I should go to the operating room. I wanted someone to take my hand and say my baby was fine, but nobody even looked at me. All the anxiety came over me there just because they said that the baby might die”*

Several strategies were mentioned to improve staff’s behavior with mothers:

*“My midwife was an angel. I was wondering why she did not get tired. If I had asked her a hundred times, she would take my hand to get up and she was so kind. I think all my good memories of my childbirth are due to my kind midwife”.*

Some codes suggested that fear and anxiety would be far from the mother if she had deep trust in her caregiver. Caregivers should therefore adopt approaches that can strengthen the mother’s trust during pregnancy, throughout e.g. responding to their needs:

“*I did not know my doctor well. I did not have faith in her. I was always afraid that she would make a mistake during the operation. If I really believed in her, everything would have been different. I would have given myself to her in the operation room, but I could not, and everything was ruined”.*

Some interviewees argued that medicalization of maternity can turn childbirth into a bitter experience. Emergency cesarean sections following unnecessary medical interventions during labour was one of the main causes of PBT in this study. Such an emergency situation was perceived to be intolerable for an unprepared, unsupported, tired, and defeated woman. Promoting physiologic childbirth was identified as an important strategy in preventing PBT:

*“The augmentation is a routine intervention for all mothers as soon as they begin labour. Physiologic childbirth does not make sense in Iran. Unnecessary interventions impede labour progress and causes complication. The result is emergency cesarean section and exposure to trauma will be therefore inevitable”.*

Specific strategies for mental healthcare services* are such that the health system should consider measures that can identify individuals with high-risk mental problems during pregnancy, so caregivers will be able to provide some special interventions for those individuals. Setting up clinical standards to maintain and enhance mental health* was another effective measure that was mentioned to take:

“*When a series of standards make the staff identify specific people, and screening is done smoothly... For instance, people who have delivery phobias. Well, they can be treated during pregnancy. Otherwise, the mother will be traumatized*”.

Labour pain relief was an issue related to the theme responsible caregiving that was frequently mentioned in our study. Many expert participants were of the opinion that pain reduction methods, i.e. epidural anesthesia, cannot prevent the development of PBT on their own. Rather, accepting the labour pain was mentioned to be an effective way for preventing PBT:

“*You see, plastic surgeries are very painful, aren’t they? Are there few of them? If a person accepts it, she would accept it well. In my opinion, awareness of this process and accepting it, would be much more important than merely painless process”.*

### The alliance of prenatal and antenatal care

The inconsistency between prenatal care goals and childbirth care was among the most important identified problems. Our literature review identified two strategies that were developed during interviews’ analysis. Seven experts proposed continuous care by one person or a specific care team from the beginning of pregnancy until childbirth, as an effective solution for preventing PBT:

“*Continuity of care is the solution... This is the best that in our country can reduce the fear of childbirth and pregnancy concerns, as well as mental health problems at birth. Midwifery led care and the continuing midwifery support are the development that must be created to reduce maternal mental problems”.*

This strategy may not be applicable in some health systems, hence alternative methods such as national electronic prenatal-care records systems* were proposed for coordinating caregivers:

*“The electronic health record that is close to implementation can be helpful. The mother feels that her care is not inseparable. Wherever she goes, there is a code that reveals the entire records of her pregnancy with the details needed*”.

### Reconstruction of the structures

The strategies under this theme were extracted directly from the interviews and included changes in the management system or the environment to improve maternity care. These solutions are prerequisites for the best execution of other themes and include: adoption of new laws*, monitoring the implementation of laws*, financing personnel*, the use of human resources*, appropriateness of the description of tasks with the capacity of labor*, and the renewal of labour wards*.

Some suggested that new laws should include “increasing the autonomy of the midwives”, “hospitals’ grading based on their quality of care”, and “fair-wage pay for midwifery care”. They were of the view that these rules may lead to positive competition among healthcare providers, increase motivation for providing dignified care, reduce medical interventions during labour, and succeed implementation of midwifery-led care models.

In addition, many mothers expressed their fear of facing the hospital environment, for instance the color of walls and curtains in the delivery rooms, improving which can contribute to the ideal conditions for mothers’ mental health:

*“As a mother, I prefer warm colors to be used in hospital wards, because it is very important for me, for example, that instead of metal furniture the furniture of the childbirth room be wooden to give warmth. The current environment is not good for the birth room. Dumb colors are everywhere”*

One father interviewee said: *“It is awful that women do not have a private environment for labour, so the hospitals need to be rebuilt and everyone have their own room. I think nobody pays attention that the childbirth environment should be charming, cozy, and warm*”.

## Discussion

This qualitative study aimed to identify and explain the primary strategies for preventing PBT. Our review identified no study that directly deals with prevention of PBT. Our findings revealed four general areas: training, care reform, the uniformity of maternity care and infrastructure reform, which were broken down into several interventions to improve mothers’ mental health and reduce the psychological damage of childbirth.

We asked mothers experiencing PBT ‘What should we do to not be traumatized?’. Our findings complements the previous study looking for PBT causes [11]. Examining the existing interventions and health policies helped researchers provide the most complete spectrum of potential strategies for preventing PBT. Finally, the clinical opinions of health experts added some operational strategies to the list. Our lessons learned can guide, we envisage, future efforts to reduce PBT at the clinical level, which will potentially improve women’s physical, social and emotional health [8].

Although our systematic search found no previous study with similar aims, a review study describes postnatal PTSD (not PBT) prevention strategies at three primary, secondary and tertiary levels. First-level intervention is the screening of the mother’s mental problems during pregnancy, whose high-risk cases should receive special care and support during the labor. Our study identified a similar strategy, so-called ‘Responsible Caregiving’ for preventing PBT. Secondary and tertiary prevention refers to screening and early interventions for PTSD through counseling [15], whose efficiency has been questioned in a systematic review [16]. Therefore, we did not consider them in the present study.

Many identified strategies are used in various countries as part of a routine care. Nevertheless, most strategies are not integrated or implemented in less than optimal conditions, which is rare in low-middle income countries (LMICs), where staff have insufficient skill mix and low number of midwives hurdles the implementation [17]. As targeting multiple areas to improve service quality will reduce morbidity burden [7, 10], it is recommended that each country, based on its own platform, uses the strategies of each of the four domains simultaneously.

In Iran, where the prevalence of PBT is higher than the global average (54.5%) [4], most of these strategies are not widely used. Mothers might not be well-prepared for labour, medicalization is common among caregivers, no companions are allowed in many labour wards, prenatal care is entirely separate from childbirth care, and clinical monitoring and evaluation are not perfect. Few of PBT strategies are available in some public and private health centers in Iran, which may benefit from major reforms. This might be inevitable, because most birth preparation courses in Iran are not skill generating and deal only with physiology of childbirth.

According to experts, responsible caregiving strategies are probably the most effective ways to reduce PBT. What World Health Organization (WHO) referred to as ‘Quality of Care for pregnant women’ has a major overlap with the ‘responsible caregiving’ described in this study, meaning provision of high quality clinical care based on effective communication, respect, reverence and emotional support to the mother [10]. Supporting mothers during childbirth is one of the strongest strategies that can be implemented into the health system at the lowest cost [18]. An effective support in labor has the following features: strong and continuous, carried out by trained people close to the mother, and available from the beginning of the labor [19]. A recent effort by the WHO to develop respectful maternal care standards shows the global importance of staff abusive practices in maternity hospitals [10]. The elaboration of the clinical guidelines for respectful maternity care is the least a health system has to do [10]. Appropriate implementation of these guidelines requires robust evaluation and feedback mechanisms in each health system [20], such as the management strategies described under the ‘reconstruction of the structures’ theme. Medicalization of childbirth may harm responsible caregiving. A high-quality care uses all medical technologies, when needed, to perform a safe delivery [10], while it avoids unnecessary medical interventions that can exacerbate maternal stress as much as possible [19]. Although responsive caregiving strategies are often related to labour care, mental health promotion strategies, such as screening and identifying mothers at risk for mental illness, are among helpful interventions to be integrated in the Maternity and Child Health services (MCH). Providing such mental health services in prenatal visits is one of the main challenges of health systems. Development of screening and referral services for mothers with mental problems in pregnancy is recommended [21].

Strategies of skill-builder knowledge theme were developed with more focus on improving mother’s skills during childbirth. Prenatal education can be skill-generating, if it can enhance the mother’s confidence in her ability. The mother must understand the natural process of childbirth and ensure her inherent ability to give birth. She must be trained in the proper techniques of strengthening and relaxing during the labour, the ability to make decisions, trust her body and behave cleverly in critical situations. Thus, childbirth courses should not be limited to the training of labour and childbirth physiology. Successful courses are the ones that focus on improving health behaviors during pregnancy, reducing stress, improving family relationships, empowering the mother, promoting mother’s confidence and skills related to baby care [22].

Besides training the pregnant woman, maternal caregivers should also be trained in care for the mother’s mental health. Strengthening the mental dimension in the curriculum of medical and midwifery students will be helpful in this regard. Caregivers must learn appropriate skills to communicate effectively with the mother at various stages of the labour, how to inform the mother and her companion, and help the mother participate actively in childbirth. Professionals and midwives must understand the importance of focusing on the mothers’ mental and emotional needs from the start of their education and get to know that they directly affect the long-term experiences of mother’s birth [23, 24]. In order for training interventions to lead to change in the behavior of clinicians and maternity experts, continuous training, monitoring and feedback, participation of supervisors, academic literacy and periodic reminders should be utilized [25].

The purpose of the alliance of prenatal and antenatal care strategies is to ensure that the provider of labour care can effectively communicate with the mother to prevent the occurrence of mental trauma. For example, if prenatal care providers, through the insertion of warning signs on the mother’s record, inform the labour caregivers of the mother’s mental status, the occurrence of PBT will decrease [26]. Midwifery-led care will enhance maternal satisfaction with all aspects of care. The use of this care model depends on the health policies of the countries, midwives’ autonomy, and how maternity care structures are shaped. It seems that the implementation of this care approach is highly cost-effective and reduces other maternal morbidities. e.g. preterm labour [27].

### Study limitations

As with other qualitative studies, findings cannot be generalized to the community. Another limitation is that the social and cultural factors involved in personal experiences can affect the results. Sampling with maximum diversity was used to reduce these limitations. During the comprehensive review of international resources, we attempted to diminish the effect of personal experiences on final strategies. Moreover, the strategies derived from the review of evidence guided the interviews. Our research identified some information gaps, so we conducted interviews to complete the gaps and expand the existing knowledge. We strongly recommend pilot and trial studies to determine the effect of these potential strategies on PBTs.

## Conclusions

Implementation of targeted PBT prevention interventions is recommended as a part of maternity care for promotion of maternal mental health. Identification of the domains and interventions is the first step in this regard. This study identified the existing and potential strategies for preventing PBT and categorized them within four themes of effective intervention: skill-builder knowledge, responsible caregiving, the alliance of prenatal and antenatal care and reconstruction of the structures. Each theme consists of the strategies to be implemented in the health systems. Our findings may provide a comprehensive and scientific approach for prevention of PBT for health service researchers and policy makers. We propose simultaneous implementation of multiple contextual-based strategies for reduction of PBT to get the highest efficiency. Prioritization of these strategies should be, we recommend, based on the collective views of policymakers in the health system of each country. Pilot studies are necessary to evaluate resources and modify approaches, prior to national rule out of PBT prevention strategies.

## Data Availability

The datasets generated and analyzed during the current study are not publicly available due to considerations of confidentiality but anonymized data are available from the corresponding author on reasonable request.

## Abbreviations

PBT: Psychological Birth Trauma
PTSD: Post Traumatic Stress Disorder
DSM-V: Diagnostic and Statistical Manual of Mental Disorders, 5th edition
CS: Cesarean Section
NVD: Normal Vaginal Delivery
NGO: Non-governmental Organization
MOHME: Ministry of Health and Medical Education
